# Evaluation of a streamlined sentinel lymph-node imaging protocol in early-stage oral cancer

**DOI:** 10.1007/s12149-021-01677-6

**Published:** 2021-09-13

**Authors:** Michiel Zeeuw, Rutger Mahieu, Bart de Keizer, Remco de Bree

**Affiliations:** 1 Department of Head and Neck Surgical Oncology, University Medical Center Utrecht, Heidelberglaan 100, 3584 CX Utrecht, The Netherlands; 2 Department of Radiology and Nuclear Medicine, University Medical Center Utrecht, Heidelberglaan 100, 3584 CX Utrecht, The Netherlands

**Keywords:** Mouth neoplasms, Lymphatic metastases, Sentinel lymph-node biopsy, Lymphoscintigraphy, Single photon emission computed tomography computed tomography

## Abstract

**Objective:**

Sentinel lymph-node (SLN) mapping for early-stage oral squamous cell carcinoma (OSCC) is comprehensive and consequently time-consuming and costly. This study evaluated the clinical value of several SLN imaging components and analyzed the accuracy for SLN identification using a streamlined SLN imaging protocol in early-stage OSCC.

**Materials and methods:**

This retrospective within-patient evaluation study compared both number and localization of identified SLNs between the conventional SLN imaging protocol and a streamlined imaging protocol (dynamic lymphoscintigraphy (LSG) for 10 min directly post-injection and SPECT-CT at ~ 2 h post-injection). LSG and SPECT-CT images of 77 early-stage OSCC patients, scheduled for SLN biopsy, were evaluated by three observers. Identified SLNs using either protocol were related to histopathological assessment of harvested SLNs, complementary neck dissection specimens and follow-up status.

**Results:**

A total of 200 SLNs were identified using the streamlined protocol, and 12 additional SLNs (*n* = 212) were identified with the conventional protocol in 10 patients. Of those, 9/12 were identified on early static LSG and 3/12 on late static LSG. None of the additionally identified SLNs contained metastases; none of those in whom additional SLNs were identified developed regional recurrence during follow-up. Only inferior alveolar process carcinoma showed a higher rate of additionally identified SLNs with the conventional protocol (*p* = 0.006).

**Conclusion:**

Early dynamic LSG can be reduced to 10 min. Late static LSG may be omitted, except in those with a history of oncological neck treatment or with OSCC featuring slow lymphatic drainage. Early static LSG appeared to be contributory in most OSCC subsites.

## Introduction

Sentinel lymph-node (SLN) biopsy has been studied widely and has proven to be reliable in staging the clinically negative neck in early-stage OSCC patients, with a pooled sensitivity and negative predictive value of 87% and 94%, respectively [1–5]. Today, SLN biopsy is implemented as standard oncological care in nearly all Dutch head and neck oncological centers for staging the clinically negative neck in patients with early-stage OSCC [6].

In essence, SLN mapping is initiated by peritumoral injections of a ^99m^Tc-labeled radiotracer. Directly post-injection planar dynamic and early static lymphoscintigraphic (LSG) imaging is acquired, followed by late static LSG and SPECT-CT imaging [7–10].

The current SLN imaging protocol in early-stage OSCC is comprehensive. Accordingly, it is associated with high costs, patient discomfort and limited availability of SPECT-CT imaging devices on day-to-day basis [11]. There may be opportunities to develop a protocol which is less costly and time-consuming for both medical professionals and patients.

In 2012, Heuveling et al. already underlined the limited value of late static LSG in early-stage OSCC. The authors stated that late static LSG is only contributory in selected cases and should not be routinely performed [12]. This finding is in concordance with practice guidelines of Alkureishi et al. suggesting that late static LSG should only be performed if early static LSG does not depict any hotspots [8]. More recently, the European Association of Nuclear Medicine (EANM) revised their guidelines by advising that early dynamic LSG should encompass the first 10–15 min post-injection, instead of 30 min as performed in our institution [9].

In an attempt to streamline the current SLN imaging protocol, this study evaluated the clinical relevance of several routinely performed SLN imaging components, as performed in our institution. Furthermore, this study compares the accuracy for SLN identification using a streamlined SLN imaging protocol with the conventional SLN imaging protocol, in early-stage OSCC patients.

## Materials and methods

### Ethical considerations

This study abided the Declaration of Helsinki and was approved by the Ethics Committee (no. 19-397). Requirement for informed consent was waived by the Internal Review Board. Pathological, imaging and clinical data were dealt with in accordance to General Data Protection Regulation.

### Patients

Patients with early-stage OSCC (cT1-3N0), who underwent SLN biopsy in our institution between December 2017 and March 2020, were included in this study (AJCC UICC TNM-staging 8th Edition) [13, 14]. Patients with a primary tumor staged cT3 were only included when tumor dimensions ≤ 4 cm [13]. Clinical nodal staging was confirmed by at least ultrasound; ultrasound-guided fine needle aspiration cytology was performed in case of suspected lymph nodes.

Patients were excluded if the administered dosage in megabecquerel (MBq) was not in line with the most recent guidelines of the EANM [9].

### Sentinel lymph-node imaging procedure

All patients underwent planar static and dynamic LSG and SPECT-CT imaging the day prior surgery (2 day protocol) or the day of surgery (single-day protocol) on a Siemens Symbia T16 SPECT-CT scanner, using ‘low- and medium energy’ (LME) collimators. A total of 2–4 peritumoral injections were administered with ^99m^Tc-labeled radiotracer (i.e., nanocolloid, tilmanocept). For the 2 day protocol, ~ 120 MBq (3.24 mCi) [^99m^Tc]Tc-nanocolloid or ~ 74 MBq (2.0 mCi) [^99m^Tc]Tc-tilmanocept was administered, whereas for the single-day protocol, ~ 50 MBq (1.35 mCi) [^99m^Tc]Tc-nanocolloid was administered. Directly post-injection planar dynamic LSG was acquired in anterior view (128 × 128 matrix; 60 frames of 30 s). Then, early planar static LSG was acquired in anterior view (256 × 256 matrix; 240 s) and anterior-oblique view from both sides (256 × 256 matrix; 480 s), with additional Co-57 flood source images (3 × 30 s) for contour detection. 3D SPECT-CT was acquired at 90–120 min post-injection, for a total duration of 35 min, on a 128 × 128 matrix (pixel spacing, 3.9 × 3.9 mm), with 128 angles, 20 s per projection, over a non-circular 360° orbit (CT:110 kV, 40 mAs eff., 16 × 1.2 mm). SPECT images were reconstructed using clinical reconstruction software (Siemens Flash3D), with attenuation and scatter correction (6 iterations, 8 subsets, 5 mm Gaussian filter). SPECT-CT imaging was immediately followed by late planar static LSG with flood field images (256 × 256 matrix; 3 × 240 s, 3 × 30 s) [15]. Identified SLN(s) were marked on the corresponding overlying skin with a Co-57 pen point marker.

### Surgery, histopathological assessment and follow-up

Intraoperatively, the marked SLN(s) were localized under at least handheld gamma probe guidance, accompanied by surgical extirpation. Extirpated SLN(s) were subjected to histopathological assessment according to SLN biopsy protocol (i.e., step-serial-sectioning, hematoxylin–eosin staining and immunohistochemistry) [8, 16]. For those in whom SLN(s) were negative for metastasis, a wait-and-scan policy was adopted. SLN biopsy-positive patients, however, underwent complementary treatment of the neck [i.e., neck dissection and/or (chemo) radiotherapy]. Complementary neck dissection specimens were routinely assessed for additional lymph-node metastases by histopathological examination. Follow-up visits were scheduled according to standard oncological care.

### Streamlined imaging protocol

**Fig. 1:**
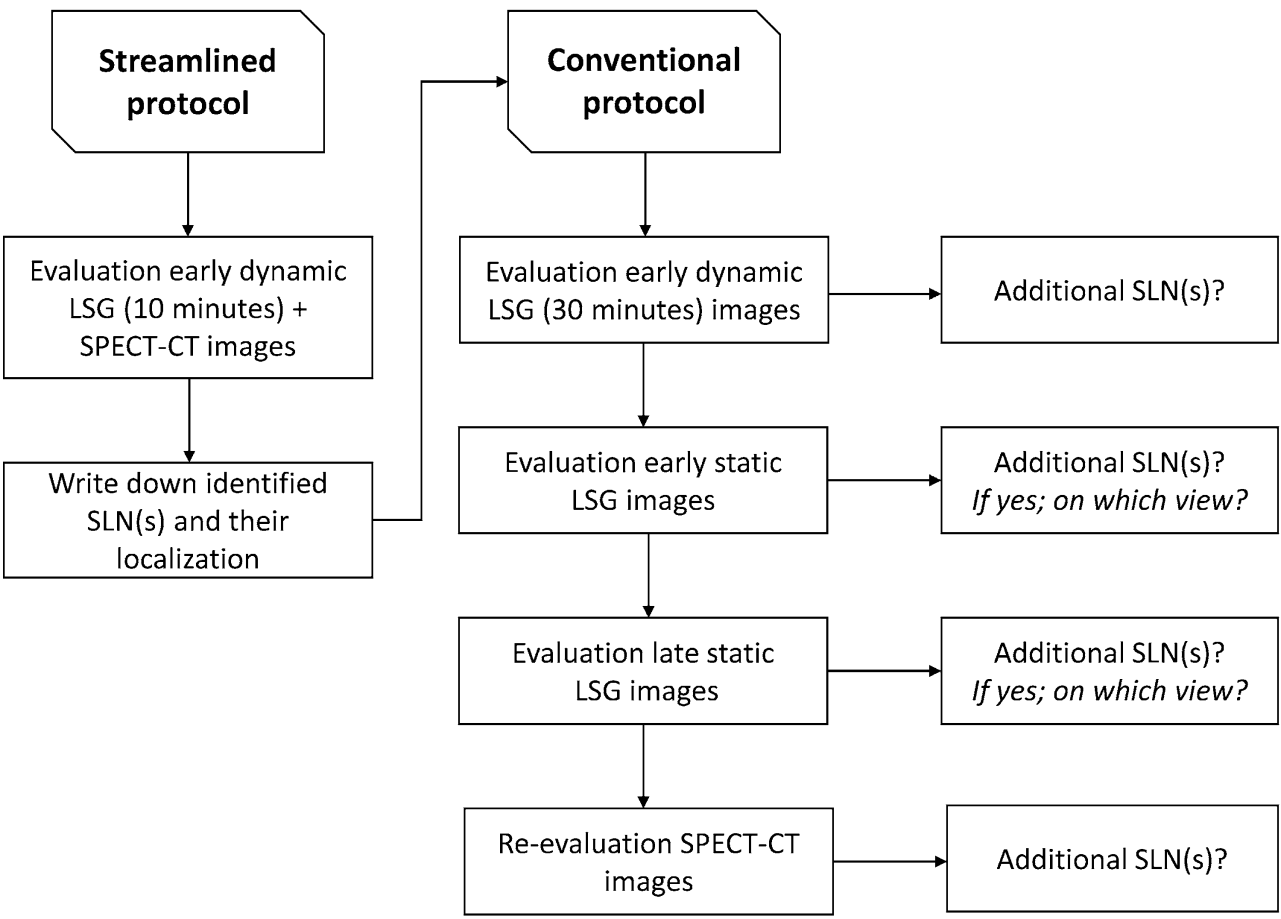
*LSG* lymphoscintigraphic, SLN sentinel lymph node

For the streamlined protocol, selected conventional imaging components were omitted or its acquisition time was reduced (Table 1; Fig. 1). Planar dynamic LSG was reduced to 10 min. Then, SPECT-CT images, acquired ~ 2 h post-injection, were analyzed. Both early and late planar static LSG were omitted for the streamlined protocol.

**Table 1:** Components and duration of the conventional and streamlined SLN imaging protocol

	Conventional protocol	Streamlined protocol
Early planar dynamic LSG; in minutes	30:00	10:00
Early planar static LSG; in minutes		
Anterior view	4:30	Omitted
Anterior-oblique view L+R	9:00	Omitted
SPECT-CT; in minutes	35:00	35:00
Late planar static LSG; in minutes		
Anterior view	4:30	Omitted
Anterior-oblique view L+R	9:00	Omitted
Total; in minutes	92:00	45:00

*SLN* sentinel lymph node, *LSG* lymphoscintigraphy, *L* left, *R* right

### Evaluation

Both imaging protocols were evaluated retrospectively by 3 observers with considerable experience in analyzing LSG and SPECT-CT images for SLN mapping in early-stage OSCC (Fig. 1). In case of discrepancies between observers as a joint team deciding unanimously, consensus was obtained through discussion.

First, the observers were asked to classify visualized lymph nodes as ‘yes’, ‘no’ or ‘potential’ as to being SLNs based on the streamlined protocol. Subsequently, all lymph nodes classified as ‘potential’ had to be labeled ‘yes’ or ‘no’, indicating the advice on its surgical extirpation.

Second, the individual components of the conventional imaging protocol were evaluated in consecutive order. The observers were asked to identify whether additional SLNs were identified for each component. If any additional SLNs were identified, the observers were asked on which view (e.g., anterior–posterior, anterior-oblique) the additional SLNs were first identified.

To identify false-negative outcomes of the streamlined imaging protocol, identified SLN(s) using either imaging protocol were related to histopathological status of harvested SLNs. Furthermore, in case of additional lymph-node metastases in complementary neck dissection specimens, their corresponding location was correlated to images from either imaging protocol. Finally, for those who developed regional nodal recurrence during follow-up, the corresponding location of the regional nodal recurrence was correlated to images from either imaging protocol as well. Regional nodal recurrences in presence of local tumor recurrence or second primary tumors were not considered suitable for such correlation, since differentiation between missed occult nodal metastasis by SLN biopsy and metastasis developed from reseeding tumors is unfeasible. Regional nodal recurrences that occurred in a side of the neck which was initially staged positive for nodal metastasis by SLN biopsy were not considered false-negative outcomes, as such a regional recurrence is considered to be a consequence of insufficient complementary treatment rather than inadequate SLN biopsy.

### Statistical analyses

All data were analyzed with IBM SPSS Statistics Version 26.0. For categorical variables, the number of cases and its percentage were calculated. Continuous parametric variables are presented as mean (± SD), whereas non-parametric variables are presented as median with interquartile range (IQR).

To assess whether not identifying all SLNs with the streamlined protocol was associated with patient, tumor or imaging characteristics, univariate analyses were applied. Independent Samples *t* test was applied for parametric continuous variables (i.e., tumor size) and Mann–Whitney *U* test for non-parametric continuous variables (i.e., DOI and administered radioactive dosage). For categorical variables (i.e., tumor localization, clinical T-stage and used radiotracer, Chi-square tests were applied; in case of variables with small samples (*n* ≤ 5), Fisher’s exact test was used (i.e., midline involvement and 1-/2-day imaging protocol). In case of significant association for categorical variables with  ≥ 3 groups, subsequent post hoc analyses were conducted.

Finally, Spearman’s rank-order correlation was used to assess the association between both amount as well as location of identified SLNs per patient with both SLN imaging protocols.

A *p* value ≤ 0.05 was considered to be statistically significant.

## Results

Out of a total of 92 patients, 77 patients were included in this study. In those excluded (*n* = 15), the administered dosage in megabecquerel (MBq) was not in line with the most recent guidelines of the EANM [9].

Of those included in this study, 9 (11.7%) had undergone previous neck treatment for head and neck malignancies (Table 2). Of all primary tumors, the majority was located in the tongue (59.7%). Most patients presented with tumors clinically staged T1- or T2 (92.2%). SLN biopsy showed cervical lymph-node metastases in 19 (24.7%) patients. Mean follow-up time after surgery was 14.9 (± 7.2) months.

**Table 2:** Patient and tumor characteristics

Characteristics	*N* = 77
Gender	
Male (%)	45 (58.4%)
Female (%)	32 (41.6%)
Age at scan; mean (± SD) in years	62.5 (± 13.0)
Previous neck treatment	
None (%)	68 (88.3%)
Neck dissection (%)	4 (5.2%)
Radiotherapy (%)	3 (3.9%)
Neck dissection and chemoradiation (%)	2 (2.6%)
Anatomical localization primary tumor	
Tongue (%)	46 (59.7%)
Floor-of-mouth (%)	14 (18.2%)
Buccal mucosa (%)	8 (10.4%)
Retromolar area (%)	5 (6.5%)
Inferior alveolar process (%)	4 (5.2%)
Clinical T-stage primary tumor	
cT1 (%)	31 (40.3%)
cT2 (%)	40 (51.9%)
cT3 (%)	6 (7.8%)
Pathological N-stage	
N0 (%)	58 (75.3%)
N1 (%)	8 (10.4%)
N2a (%)	1 (1.3%)
N2b (%)	5 (6.5%)
N2c (%)	3 (3.9%)
N3b (%)	2 (2.6%)
Follow-up time; mean (± SD) in months	14.9 (± 7.2)

TNM-staging according to AJCC UICC 8th Edition [12, 13]

*SD* standard deviation

**Fig. 2:**
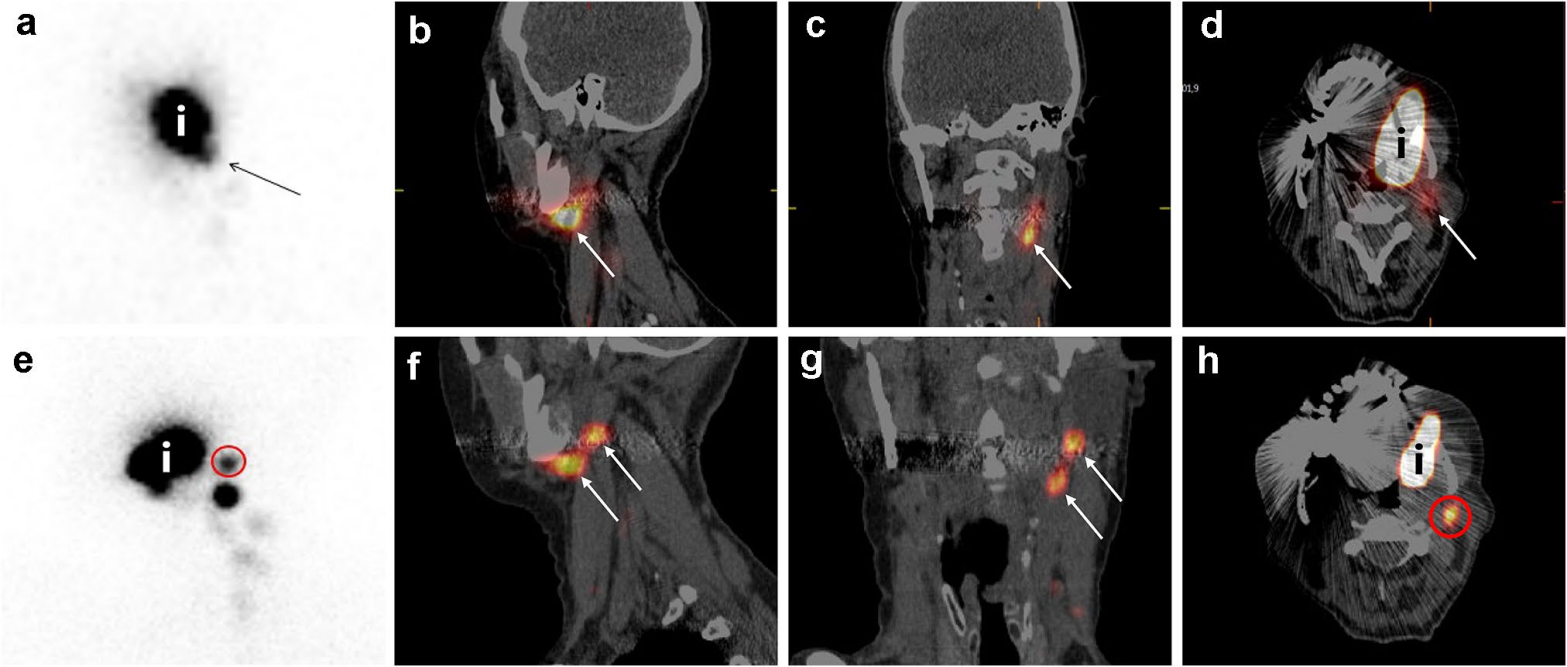
A 77 year old patient (patient 3) with a cT2N0 primary tumor in the inferior alveolar process, located on the left side. Using the streamlined protocol, two SLNs were identified (level Ib left; level IIa left). Evaluation of early static LSG (anterior-oblique view) allowed discrimination between two hotspots in level IIa. Written informed consent for publishing these images was obtained from this patient. **a** Planar early dynamic LSG 10 min anterior view; one hotspot level Ib left (arrow). **b** SPECT-CT sagittal plane; one large hotspot level IIa (arrow). **c** SPECT-CT coronal plane; one large hotspot level IIa left (arrow). **d** SPECT-CT axial plane; injection site (i) and one large hotspot level IIa left (arrow). **e** Planar early static LSG anterior-oblique view; one additionally identified hotspot level IIa left (red circle). **f** Post-evaluation SPECT-CT reconstruction* sagittal plane; discrimination between two hotspots level IIa (arrows). **g** Post-evaluation SPECT-CT reconstruction* coronal plane; discrimination between two hotspots level IIa left (arrows). **h** Post-evaluation SPECT-CT reconstruction* axial plane; more cranially localized one additionally identified hotspot (red circle). *SPECT-CT reconstructions were made with ITK-SNAP (www.itksnap.org) [17]. *LSG* lymphoscintigraphy

A total of 200 SLNs were identified using the streamlined imaging protocol; 12 additional SLNs (thus in total 212) were identified with the conventional imaging protocol in 10 patients (Table 3). Figure 2 illustrates how an additional SLN was identified based on early static LSG (anterior-oblique view).

**Table 3:** Patients with additionally detected SLNs based on conventional imaging components

Patient	Primary tumor	SLNs on streamlined protocol	PA	Additionally identified SLNs	Based on which image	PA extra SLN	Follow-up time (months)	Follow-up status
1	Tongue R	IIa R	−	IIb R	Early static oblique	−	22	NED
		III R	−					
		III L	n.s.r	IIb R	Early static oblique	−		
		IV R	n.s.r					
2	Buccal mucosa L	III R	−	Va L	Early static AP	n.s.r	21	NED
3	Inferior alveolar process L	Ib L	n.s.r	IIa L	Early static oblique	−	11	Local recurrence
		IIa L	+					
4	Inferior alveolar process R	None		IIa R	Early static oblique	−	21	NED
				Ib R	Late static oblique	−		
5	Buccal mucosa L	Ib L	+	IIa L	Early static oblique	n.s.r	19	NED
6	Tongue R	IIa R	−	Ib R	Early static oblique	n.s.r	16	NED
		III R	−					
7	Tongue L	IIa L	−	Ib L	Early static oblique	−	15	NED
8	Retromolar area R	IIa R	−	IIa R	Late static oblique	−	11	NED
9	Floor-of-the-mouth R	III R	−	IIa R	Early static oblique	−	11	NED
		III L	−					
10	Inferior alveolar process L	Ia L	−	III L	Late static oblique	n.s.r	5	NED
		III L	−					

*SLN* sentinel lymph node, *PA* histopathological assessment, *R* right side, *L* left side, *n.s.r.* not surgically removed,  +  histopathologically positive, − histopathologically negative, *NED* no evidence of disease

Of the 12 additionally identified SLNs, 9 (75%) were identified on early static LSG and 3 (25%) on late static LSG. All hotspots visualized during early static LSG remained visible on late static LSG. In the 3 patients in whom additional SLNs were identified on late static LSG only, 2 primary tumors were located in the inferior alveolar process and 1 in the retromolar area. The added 20 min early dynamic LSG of the conventional protocol did not allow identification of additional SLNs.

None of the additionally identified SLNs were histopathologically positive. In addition, none of the patients in whom additional SLNs were identified by the conventional SLN imaging protocol developed regional nodal recurrence after a mean follow-up of 15.2 (± 5.6) months. Univariate analyses showed that only the primary tumor site was associated with not identifying all SLNs using the streamlined protocol (*p* = 0.002). Post hoc analyses showed that only inferior alveolar process carcinoma was associated with not identifying all SLNs using the streamlined protocol (*p* = 0.006) (Table 4).

**Table 4:** Are all SLNs identified with the streamlined SLN imaging protocol?

*n* = 77	Yes (*n* = 67)	No (*n* = 10)	*p* value*
Site of primary tumor			**0.006**†
Tongue (%)	43 (93.5%%)	3 (6.5%)	
Floor-of-mouth (%)	13 (92.9%%)	1 (7.1%)	
Buccal mucosa (%)	6 (75.0%)	2 (25.0%)	
Inferior alveolar process (%)	1 (25.0%)	3 (75.0%)	
Retromolar area (%)	4 (80.0%)	1 (20.0%)	
Tumor size; mean (± SD) in mm	19.07 (**± **5.42)	18.67 (**± **7.26)	0.873
DOI; median (IQR) in mm	7.00 (4.00)	6.00 (3.00)	0.478
Midline involvement primary tumor			0.343
Yes (%)	10 (100.0%)	0 (0.0%)	
No (%)	57 (85.1%)	10 (14.9%)	
cT-stage			0.773
T1 (%)	28 (90.3%)	3 (9.7%)	
T2 (%)	34 (85.0%)	6 (15.0%)	
T3 (%)	5 (83.3%)	1 (16.7%)	
Imaging protocol			0.638
1 day (%)	6 (85.7%)	1 (14.3%)	
2 day (%)	61 (87.1%)	9 (12.9%)	
Radiotracer used			0.495
[^99m^Tc] Tc-nanocolloid (%)	49 (84.5%)	9 (15.5%)	
[^99m^Tc] Tc-tilmanocept (%)	18 (94.7%)	1 (5.3%)	
Dosage used; median in MBq (mCi)	116.00 (3.14)	121.50 (3.28)	0.282
Previous neck treatment			0.088
None (%)	61 (89.7%)	7 (10.3%)	
Neck dissection (%)	2 (50.0%)	2 (50.0%)	
Radiotherapy (%)	2 (66.7%)	1 (33.3%)	
Neck dissection and chemoradiation (%)	2 (100.0%)	0 (0.0%)	

TNM-staging according to AJCC UICC 8th Edition [13, 14]

*SD* standard deviation, *DOI* depth-of-invasion, *IQR* interquartile range, *MBq* megabecquerel, *mCi* millicurie

*Bold if statistically significant

†Significance regards inferior alveolar process

In 3/4 (75%) patients with inferior alveolar process carcinoma, the streamlined SLN imaging protocol did not allow identification of all SLNs. In those 3 patients, 4 additional SLNs were identified. In one of those patients, no drainage was observed with the streamlined SLN protocol; however, the conventional SLN imaging protocol allowed identification of 2 SLNs with marginal activity. In the remaining 1/4 (25%) patient with inferior alveolar process carcinoma, no drainage at all was observed on dynamic and static LSG and SPECT-CT images.

Out of those who underwent previous oncological treatment of the neck [i.e., neck dissection and/or (chemo) radiation], additional SLNs were identified by early static LSG in one patient (patient 2, selective neck dissection left) and on late static LSG in two patients (patient 8, bilateral neck irradiation; patient 10, selective neck dissection right).

Finally, Spearman’s showed a statistically significant correlation between identified SLNs and their corresponding location using both SLN imaging protocols for each patient *(r*_s_ = 0.898; *p* < 0.000).

## Discussion

The aim of this study was to evaluate the clinical relevance of several components of the conventional SLN imaging protocol and to assess the reliability of a streamlined SLN imaging protocol in early-stage OSCC. Using the streamlined SLN imaging protocol 200 SLNs were identified, whereas the conventional SLN imaging protocol allowed identification of 12 additional SLNs. None of the additionally identified SLNs contained metastases; none of the patients in whom they were identified developed regional nodal recurrence.

Early dynamic LSG with a duration of 30 min showed no additional diagnostic value over a duration of 10 min; no additional SLNs were identified based on evaluation of 20 supplementary minutes of dynamic LSG. This finding is in accordance with the revised guidelines of the EANM, stating that early dynamic LSG should encompass the first 10–15 min post-injection [9]. In early-stage breast cancer, dynamic LSG was completely omitted from the SLN imaging protocol, without interfering with its diagnostic accuracy, as immediate dynamic LSG had no additional value in identifying SLNs [18–21]. In early-stage OSCC, however, dynamic LSG immediately post-injection is deemed essential, as it allows visualization of lymphatic vessels draining the injection site; assisting the discrimination between SLNs and higher echelon nodes (HEN) in the complex anatomy of the neck with its abundant lymph nodes [8]. Erroneously considering HENs as SLNs induces unnecessary exploration of the neck, with its accompanying morbidity and risk of complications, that may hamper a complementary neck dissection in case of metastatic involvement of SLNs [12]. Therefore, a complete omission of early dynamic LSG is not recommended for the SLN imaging protocol in early-stage OSCC.

Early static LSG allowed for identification of additional SLNs in 8/77 (10.4%) patients. Early static LSG was contributory in nearly all OSCC subsites, as for those in whom additional SLNs were identified by early static LSG, the primary tumors were located in the tongue (*n* = 3), buccal mucosa (*n* = 2), inferior alveolar process (*n* = 2) and floor-of-mouth (*n* = 1). Even though none of these additionally identified SLNs were histopathologically positive, nor did any of these patients develop regional nodal recurrence, the omission of early static LSG may lead to a substantial rise in false-negative SLN biopsy outcomes. Moreover, differentiating either relevant SLN or irrelevant HEN can be facilitated by comparing early static LSG images to SPECT-CT images acquired at ~ 2 h post-injection. Accordingly, it is our considered opinion that one should not refrain from early static LSG acquisition for all OSCC subsites.

Late static LSG contributed for 3/12 (25%) of the additionally identified SLNs in 3/77 (3.9%) patients. Thus, out of a total of 212 SLNs, only 3/212 (1.4%) SLNs were identified on late static LSG. Heuveling et al. already underlined the limited value of late static LSG and recommended its acquisition only in patients with tumors featuring slow or limited lymphatic drainage (i.e., buccal mucosa, inferior alveolar process and soft-palate) [12]. This recommendation is mainly in concordance with our results, since in 2/4 (50%) patients with inferior alveolar process carcinoma and in 1/5 (20%) patients with tumors of the retromolar area, additional SLNs were identified on late static LSG. Furthermore, for most prevalent anatomical localizations of OSCC—tongue, floor-of-mouth and buccal mucosa—late static LSG was not contributory in our population. Of those in whom additional SLNs were identified by late static LSG (*n* = 3), two patients had a history of neck dissection or neck irradiation. Still, in one patient (patient 10), the additionally identified SLN was detected in the non-dissected neck. Nevertheless, previous treatment of the neck has been known to alter lymphatic drainage patterns and may even decelerate and impede lymphatic drainage [22]. Therefore, late static LSG could also be valuable in those who underwent previous treatment of the neck. In the study of Heuveling et al. late static LSG showed additional SLNs in half of patients with paramedian and midline tumors exhibiting bilateral drainage [12]. Nevertheless, SPECT-CT was not yet available during their evaluation. As in our population, no additional SLNs were identified by late static LSG in those with paramedian or midline tumors, late static LSG does not appear to be of additional value in these patients if SPECT-CT imaging is acquired at ~ 2 h post-injection.

Although the value of SPECT-CT imaging was not evaluated in this study, SPECT-CT imaging is deemed indispensable for SLN mapping, since it contributes significantly to SLN identification and provides enhanced anatomical orientation [23]. Previously, den Toom et al. demonstrated that the addition of SPECT-CT to planar static LSG resulted in more precise SLN detection and suggested that its beneficial properties in regard of topographical orientation lead to a safer surgical procedure for patients [23].

The major limitation of this study remains its retrospective design, making it irrevocably susceptible to bias. As included patients underwent their oncological treatment relatively recent at our institution, while observers were not blinded during evaluation, the observers might have been prone to recall bias during the evaluation. Moreover, the evaluation was not done independently by the 3 observers, but as a joint team deciding unanimously.

Furthermore, the mean follow-up duration of this population was on average 14.9 months. Although the majority (80%) of (loco)regional recurrences in patients with OSCC occur within 12 months, it is advocated to conduct a follow-up period of at least 24 months to assure that all missed occult nodal metastases have become clinically manifest [24, 25]. A blinded prospective within-patient study with longer follow-up duration would ascertain more strength of research results.

## Conclusion

The results of this study indicate that the conventional SLN imaging protocol, as employed by our institution for early-stage OSCC, can be streamlined without interfering with its diagnostic accuracy. As early static LSG appeared to be valuable in most OSCC subsites, the recommended streamlined protocol would consist of early dynamic LSG for 10 min, early static LSG and SPECT-CT imaging at ~ 2 h post-injection. In those with OSCC featuring slow or marginal lymphatic drainage (i.e., tumors involving the inferior alveolar process or retromolar area) as well as in those who underwent previous oncological treatment of the neck, late static LSG should be acquired nonetheless. Accordingly, the acquisition time for SLN mapping may be reduced from 92 to 58.5 min in the vast majority of early-stage OSCC patients. Consequently, a streamlined SLN imaging protocol may reduce the costs of SLN biopsy altogether and decrease patient discomfort, while facilitating the availability of nuclear imaging devices on a day-to-day basis. Nevertheless, on account of the retrospective nature of this study and its relatively short follow-up duration, a blinded prospective within-patient study, with longer follow-up and histological assessment as reference standard, is required before any definite conclusions can be drawn.
